# Regulation and function of CX3CR1 and its ligand CX3CL1 in kidney disease

**DOI:** 10.1007/s00441-021-03473-0

**Published:** 2021-05-19

**Authors:** Sibylle von Vietinghoff, Christian Kurts

**Affiliations:** 1 First Medical Clinic, Nephrology Section, University Clinic of the Rheinische Friedrich Wilhelms University Bonn, Venusberg Campus 1, 53127 Bonn, Germany; 2 Institute for Molecular Medicine and Experimental Immunology, University Clinic of the Rheinische Friedrich Wilhelms University Bonn, Biomedical Center II, Venusberg Campus 1, 53127 Bonn, Germany

**Keywords:** Chemokines, Glomerulonephritis, Pyelonephritis, Macrophages, Cell migration, Tubular epithelial cells, Podocytes, LPS, Diabetic nephropathy, Hyperthension

## Abstract

Attraction, retention, and differentiation of leukocytes to and within the kidney are governed by chemokines. The chemokine CX3CL1 (fractalkine) and its receptor CX3CR1 are exemplary in this regard as they are highly expressed and further upregulated in a range of kidney diseases. CX3CL1 is chiefly produced by renal endothelium and tubular epithelium, where it promotes leukocyte attraction. Recent data suggest that in addition to established soluble mediators, cellular interactions may enhance CX3CL1 expression. The receptor CX3CR1 is essential in myeloid phagocyte homing to the kidney at homeostasis, after acute cell depletion and in inflammation. CX3CR1 and its ligand are highly regulated in human kidney diseases such as IgA nephritis, systemic lupus erythematosus, and inflammatory conditions such as transplant rejection. A mechanistic role of CX3CR1 has been established in experimental models of nephrotoxic nephritis and renal candidiasis. It is debated in fibrosis. Recent publications demonstrate a role for CX3CR1^+^ myeloid cells in radio-contrast-agent and sepsis-induced kidney damage. Systemically, circulating CX3CR1^+^ monocytes reversibly increase in individuals with renal impairment and correlate with their cardiovascular risk. In this review, we discuss role and regulatory mechanisms of the CX3CL1-CX3CR1 axis in both localized and systemic effects of renal inflammation.

## Introduction

The chemokine (C-X3-C motif) Ligand 1 (CX3CL1, also known as fractalkine) (Bazan et al. 1997) binds exclusively to its G-protein coupled receptor CX3CR1 (Imai et al. 1997). CX3CL1 exists in a stalked and a soluble form and is expressed by a variety of resident cell types. Most information is available on endothelial cells (Tanaka et al. 2020). CX3CL1 is also produced by renal tubular epithelium (Cockwell 2002). The receptor is found on leukocytes, most prominently blood monocytes, phagocytes, and T cells. Since their discovery nearly 25 years ago, a wealth of data has been collected on expression levels and function of this ligand-receptor pair.

This concise review summarizes the current knowledge and recent developments regarding CX3CR1 and CX3CL1 expressing cell types and their role of in kidney disease. Given systemic CX3CL1 elevation in human CKD and in controlled murine models (Chang et al. 2014; Shah et al. 2015; Luo et al. 2019; Roy-Chowdhury et al. 2020, p.; Li et al. 2020), new discoveries regarding regulatory mechanisms and remote effects of this pathway are discussed for their relevance in kidney disease.

## Expression patterns and regulatory mechanisms

### CX3CR1

#### Expressing cell types

**Fig. 1:**
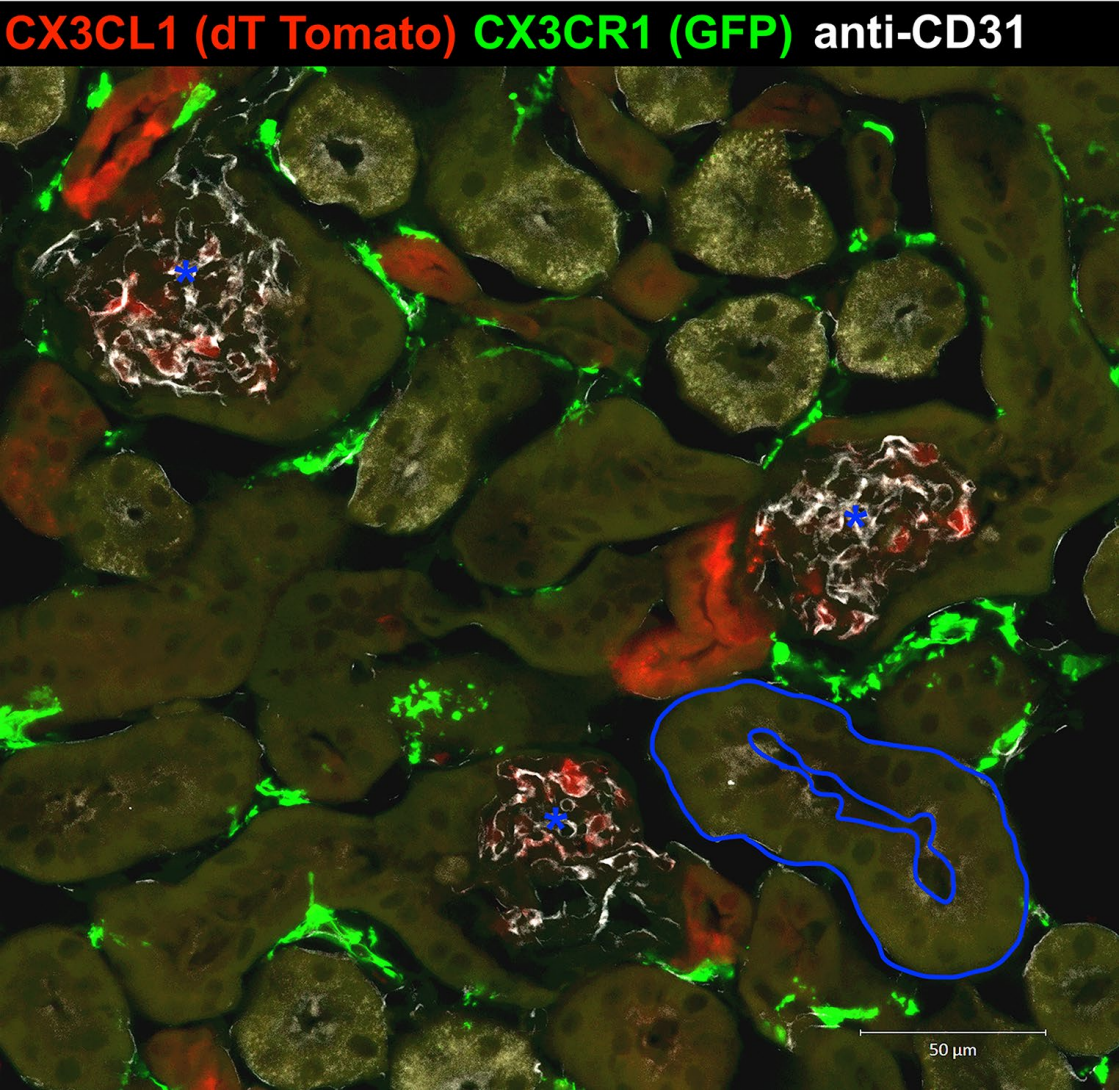
CX3CR1 and CX3CL1 expressing cells in the healthy kidney. Kidney sections of CX3CR1-GFP and CX3CL1-tdTomato double reporter mice were stained with the endothelial marker CD31 (white). Confocal microscopy demonstrated CX3CR1 reporter gene expression in interstitial cells (green) and CX3CL1 reporter gene expression (red) in glomerular endothelium (white) and tubular epithelium (size bar indicates 50 µm). Blue line indicates a renal tubule, yellow dashed lines indicate glomeruli

CX3CR1^+^ cells are abundant in the kidney. A widely distributed CX3CR1-promotor–driven GFP knock-in reporter mouse (Jung et al. 2000) has been used for the study of monocyte and phagocyte kinetics and inflammation in a range of organ systems (Tanaka et al. 2020). In the kidney, an abundant intricate tubule-interstitial network of mononuclear phagocytes was detected using this strain (Soos et al. 2006; Nelson et al. 2012) (Fig. 1). Homozygous Cx3cr1^*GFP/GFP*^ mice are CX3CR1-deficient, but cells with an active CX3CR1 promoter express green fluorescent protein and are therefore easily visualized.

Mononuclear phagocytes are among the most prominent CX3CR1 expressing cells in the kidney, most markedly on conventional type 2 dendritic cells, but also on macrophages and monocytes (Soos et al. 2006; Hochheiser et al. 2013; Stewart et al. 2019). CX3CR1 facilitated their homing to the kidney (Hochheiser et al. 2013). This was demonstrated in Cx3cr1^*GFP/GFP*^ mice, where the kidneys harbor less than 20% of dendritic cells found in Cx3cr1^*GFP/*+^ mice, especially in the cortex (Hochheiser et al. 2013). A similar dependency of the dendritic cell abundance was also observed in the intestine, but not in other organs. Human dendritic cells also required CX3CR1 for adhesion to tubular epithelium (Kassianos et al. 2015). Endothelial-mediated renal retention of monocytes in response to Toll-like receptor (TLR) 7 activation similarly depended on CX3CR1 in the mouse (Carlin et al. 2013). These findings are further strengthened by a recent publication demonstrating that CX3CR1 was essential for recovery after depletion of embryonic renal phagocytes (Liu et al. 2020). In this work, both systemic and local renal CX3CL1 levels increased after renal phagocyte depletion, which is suggestive of a regulatory circuit.

There is a recent note of caution regarding CX3CR1 expression levels on monocytes that should be considered also in renal studies: GFP-expression levels of the *Cx3cr1*^*GFP/*+^ reporter mouse (Jung et al. 2000) may not directly correlate with receptor protein expression on the surface (Meghraoui-Kheddar et al. 2020). The authors suggest to rather use the terms “classical” and “non-classical” monocytes or other markers such as Gr1/Ly6C or CD43. The investigation addressed blood and several organs but not the kidney. Also, the results do not affect the use of GFP expression levels to distinguish monocyte subtypes. They may however resolve some apparent contradictions. This includes the predominant dependence of classical monocyte tissue homing on CX3CR1, which is of relevance for the kidney (Carlin et al. 2013).

Beyond myeloid phagocytes, CX3CR1 can be expressed by diverse other renal leukocytes, including CD4^+^ and CD8^+^ T cells, NK cells, iNKT cells, and γδT cells (Cox et al. 2012; Zhuang et al. 2018). T cells have attracted most and early attention in this regard in the kidney (Cockwell 2002). However, most recent data on T cell CX3CR1 expression are from studies examining other organs. It seems to be predominantly a marker of effector cells, in both CD4^+^ (Batista et al. 2020) and CD8^+^ subtypes (Böttcher et al. 2015). Among T helper cells, prominent expression has been reported for the T helper 1 subtype (Fraticelli et al. 2001; Mionnet et al. 2010; Staumont-Sallé et al. 2014; Batista et al. 2020), especially in the lung. Effector T cells from the joints in rheumatoid arthritis also expressed CX3CR1 and bound to CX3CL1 (Sawai et al. 2005). Similarly in atherosclerosis, CX3CR1 promoted T cell homing to the aorta (Dong et al. 2016). Experiments in mixed bone-marrow chimeric mice with reduced kidney function showed that this effect was exerted in a cell individual manner. More recently, the effect of CX3CR1 on aortic T cell homing was exploited to enhance regulatory T cell homing to the atherosclerotic aorta and thereby improves atherosclerotic inflammation and lesion size (Bonacina et al. 2020).

Functional relevance of CX3CR1 for renal T cell homing remains to be explored.

#### Regulatory mediators

**Fig. 2:**
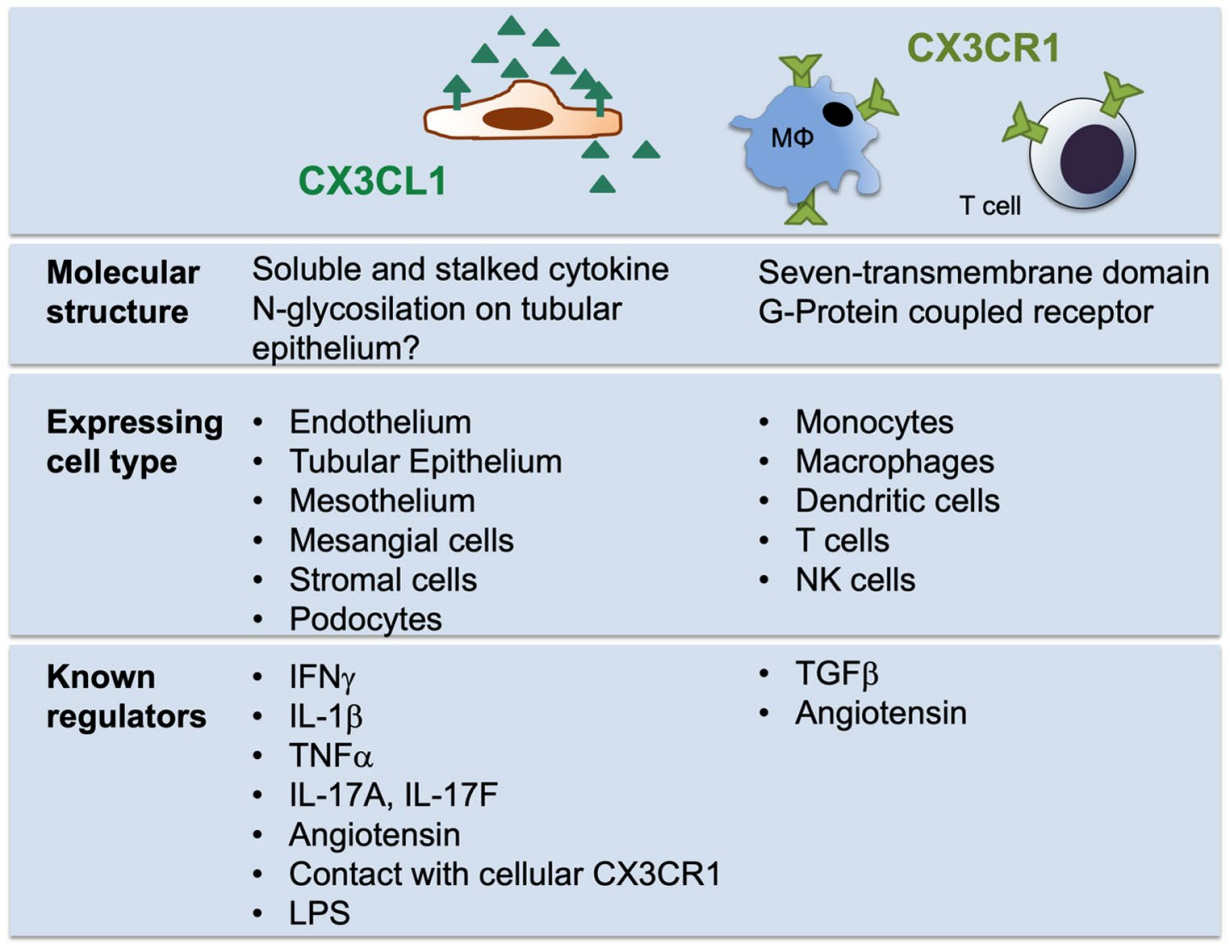
Expressional regulation of CX3CL1 and CX3CR1. The ligand CX3CL1 is known to be expressed by a variety of mostly resident cell types while CX3CR1 is characteristic of leukocytes. Mediators that have been shown to regulate their respective expression are reported

Published data on mediators that regulate CX3CR1 expression is limited and does, to the best of our knowledge, not include kidney specific studies (Fig. 2). TGFβ upregulated CX3CR1 in monocytes (Helmke et al. 2020) and T cells (Dong et al. 2016), consistent with earlier reports on microglial cells (Chen et al. 2002; Abutbul et al. 2012). Angiotensin was reported to upregulate CX3CR1 on monocyte-like cells (Apostolakis et al. 2010), consistent with recent in vivo data (Li et al. 2020). The pathophysiologic relevance of this regulation in renal disease remains to be determined.

#### Systemic regulation of *CX3CR1*^*+*^ cells in chronic kidney disease

A prominent systemic effect of severe chronic kidney disease is an increase in circulating non-classical CD16^+^, more precisely intermediate, monocytes (Heine et al. 2012). The concentration of these circulating cells in blood significantly correlates with cardiovascular events. This cell type expresses CX3CR1 on its surface. The amount of surface CX3CR1 per individual monocyte was decreased in CKD in some (Liakopoulos et al. 2018), but not other studies (Roy-Chowdhury et al. 2020). In a gene expression analysis of human monocytes, both, the non-classical monocyte marker CD16 and CX3CR1, were expressed at significantly higher levels on monocytes in patients with chronic kidney disease and cardiovascular events compared to all other groups (Schepers et al. 2015). Indeed, moderate renal impairment increased experimental atherosclerotic lesion size only in the presence of leukocyte CX3CR1 (Dong et al. 2016).

Interestingly, the increase in this cell type is reversible with kidney transplantation (Ulrich et al. 2008; Vereyken et al. 2013; Sekerkova et al. 2014; Roy-Chowdhury et al. 2020). This suggests that the phenotype is related to uremia, rather than the presence of a damaged kidney. However, specific uremic mediators that upregulate this pathway remain to be identified.

### CX3CL1

#### Expressing cell types

In the kidney, CX3CL1 expression has been reported in endothelium, tubular epithelium, mesangial cells, stromal cells, podocytes, and renal cancer cells (Zhuang et al. 2018) (Fig. 2). This includes mRNA and protein data as well as analysis of a fluorescent reporter mouse (Kim et al. 2011).

CX3CL1 expression by renal tubular epithelium has recently also been reported by single-cell mRNA analyses (the Accelerating Medicines Partnership in SLE network et al. 2019; Stewart et al. 2019). Immunofluorescence studies, but only some gene expression data, suggested predominant medullary CX3CL1 expression in the human kidney (Berry et al. 2017). A more recent single-cell study by the same group projected expression to the pelvis and connecting tubules by bioinformatical pseudodepth analysis (Stewart et al. 2019). The data remains to be confirmed by histology.

Functionally, tubular epithelium attracted monocyte-like cells, NK cells, and CD1c^+^ dendritic cells in a CX3CL1-dependent manner (Chakravorty et al. 2002; Kassianos et al. 2015). Along the same lines, recombinant CX3CL1 induced migration of CD14^+^ cells isolated from human kidneys (Berry et al. 2017). Regarding CX3CL1 on the tubular epithelial cell surface, there is one report suggesting that it is anchored via n-linked glycosylation rather than a stalk as on other cell types and immobilized on their apical surface (Durkan et al. 2007). While this remains to be confirmed by others, it should be considered when assessing the functional relevance of CX3CL1 in the complex renal architecture.

#### Regulatory mediators

CX3CL1 expression in tubular epithelium was promoted by IFNγ and TNFα (Chakravorty et al. 2002; Kassianos et al. 2015) (Fig. 2). These effects are consistent with other cell types such as endothelial cells, where TNFα, IL-1β, and LPS stimulated CX3CL1 expression (Garcia et al. 2000; Matsumiya et al. 2010; Roy-Chowdhury et al. 2020, p.). A similar action of IL-1β was reported for murine and human mesothelium (Helmke et al. 2020). In addition, both interleukin 17A and 17F strongly induced CX3CL1 in mesothelial cells. In this system and also in human endothelial cells, recent data suggest that also contact with its receptor CX3CR1 may enhance ligand expression (Roy-Chowdhury et al. 2020, p.; Helmke et al. 2020). Whether this applies to renal tubular epithelium remains to be tested.

In a more complex in vivo situation of bacterial cystitis, marked CX3CL1 upregulation was promoted by interleukin 6 in the murine bladder urothelium (Bottek et al. 2020). This may have been directly mediated or induced via liberation of messengers from other cell types. It is conceivable that this is of relevance also in the upper urinary tract.

#### Systemic CX3CL1 regulation in chronic kidney disease

A number of human studies reported elevated systemic levels of soluble CX3CL1 in CKD and a negative correlation with kidney function, including diabetic nephropathy (Shah et al. 2015) and IgA nephropathy (Luo et al. 2019). Both, reduced renal mass and enhanced production in an inflamed kidney, may contribute to these findings. The increase of serum CX3CL1 in unilaterally nephrectomized atherosclerotic mice may argue for a contribution of the former (Roy-Chowdhury et al. 2020, p.). Additionally, a very recent report describes enhanced local CX3CL1 mRNA expression in the aorta after 5/6 nephrectomy in mice (Li et al. 2020). Angiotensin receptor blockade by losartan was effective in normalization of this remote effect of severe experimental CKD, a finding that remains to be reproduced. Indeed, an earlier publication demonstrated CX3CL1 induction by angiotensin in arterial, but not venous endothelium in vivo, which increased monocyte adhesion (Rius et al. 2013).

Taken together, these data suggest the angiotensin pathway as a mechanism that elevates CX3CL1 in chronic kidney disease.

## Pathophysiologic role in kidney disease

**Fig. 3:**
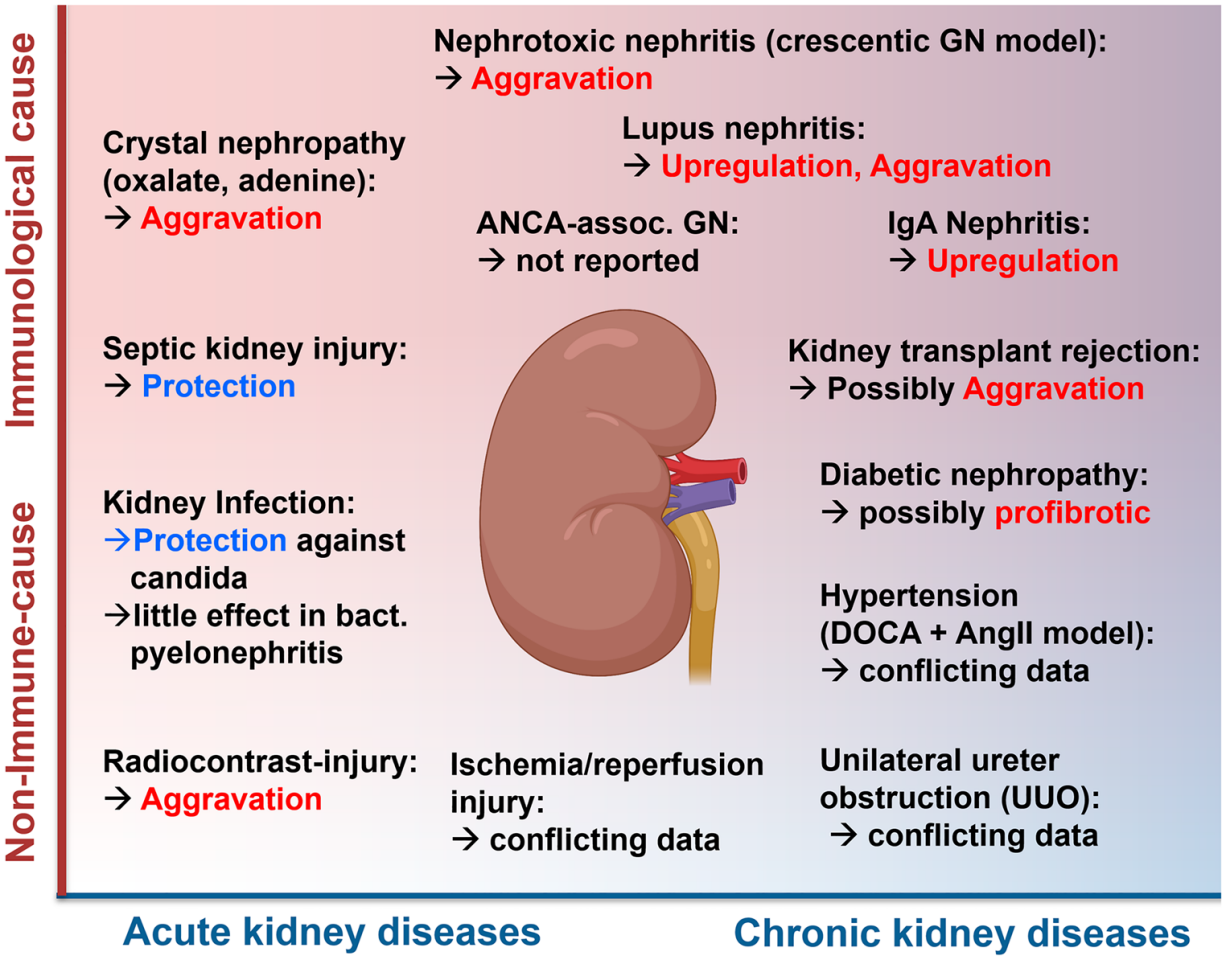
Roles of CX_3_CR1 and CX_3_CL1 in kidney diseases. Functional roles in the disease entities discussed in the review are shown. They are broadly classified into immunological and non-immune kidney diseases and by acute or chronic disease character (The kidney was drawn using Biorender®)

In humans, a seminal investigation demonstrated CX3CR1 expression in healthy adult kidneys, during development and in a variety of inflammatory conditions (Segerer et al. 2002). Since then, expression patterns in human samples and murine models of a variety of inflammatory and fibrotic kidney conditions have been detailed (Galkina and Ley 2006; Kitching 2014) (Fig. 3). We here summarize the current knowledge on expression and mechanistic relevance in kidney disease.

### Glomerulonephritides

In patients, CX3CL1 serum levels were upregulated in IgA nephritis (Cox et al. 2012; Luo et al. 2019), with a correlation of higher CX3CL1 levels with lower eGFR and higher renal inflammation assessed as CD68^+^ macrophage and CD20^+^ B cell abundance in renal biopsy tissue (Luo et al. 2019). Recent data also suggest that a similar association may exist in Henoch Schoenlein Purpura, albeit in limited patient numbers (Imai et al. 2020). The lack of a standardized murine model has yet precluded investigation of a functional role in IgA-associated renal inflammation.

While reports on ANCA-associated glomerulonephritis are lacking, both CX3CL1 and monocytes expressing CX3CR1 are known to be elevated in systemic lupus erythematosus (Nakatani et al. 2010). A more recent publication has complemented this by a detailed investigation of myeloid cells in human lupus glomerulonephritis (the Accelerating Medicines Partnership in SLE network et al. 2019). The authors studied renal biopsies from 24 patients with lupus glomerulonephritis and ten healthy controls, biopsied before living kidney donation, by single-cell mRNA sequencing. Myeloid cells were largely expanded in patients’ kidneys. Together with CXCR4, CX3CR1 was the most broadly expressed chemokine receptor on myeloid cells. However, CX3CR1 was also prominent on the predominant myeloid cell cluster of healthy kidneys. CX3CR1 expression is consistent with the fact that renal monocytic cells were rather similar to non-classical than classical monocytes. In addition, CX3CR1 was found on NK cells in healthy kidneys. This work also identified epithelial cells as main CX3CL1 producers. Currently, classification of cell clusters is still a matter of debate in single-cell analyses, especially in complex tissues such as the kidney. However, the high expression and significance levels are consistent with a wealth of protein data on CX3CR1 and its ligand (Galkina and Ley 2006; Kitching 2014).

Murine models of rapidly progressive glomerulonephritis have consistently demonstrated a pathophysiologic role of CX3CR1 and its ligand by using antibody blockade experiments (Feng et al. 1999) and CX3CR1-deficient mice (Hochheiser et al. 2013). These maneuvers reduced numbers of mononuclear phagocytes in the kidney, and thereby the inflammatory response these cells maintained. Intravital microcopy studies have revealed that CX3CR1 promotes monocyte adhesion to endothelial cells in the kidney after TLR stimulation (Carlin et al. 2013) and in antibody-mediated glomerulonephritis (Finsterbusch et al. 2016), which according to animal studies may facilitate recruitment of these dendritic cell and macrophage precursors to the inflamed kidney (Hochheiser et al. 2013). These data suggest that CX3CR1 blockade might represent a therapeutic strategy in such kidney diseases.

### Kidney transplant rejection

Among other highly inflammatory forms of kidney disease, interstitial CX3CR1^+^ positivity correlated with outcome in human transplant rejection (Hoffmann et al. 2010). Most of the interstitial CX3CR1^+^ cells were identified as macrophages and dendritic cells. Despite the fact that there was no association of recipient CX3CR1 genotype with cell infiltrates in early surveillance biopsies (Bräsen et al. 2017), this may illustrate the emerging role of macrophages in detrimental allograft inflammation (Ordikhani et al. 2020). However, animal models that precisely mirror the human situation are required for in vivo mechanistic insights in the renal alloimmune response.

### Diabetic kidney disease

CX3CR1 and CX3CL1 are upregulated in the kidney in diabetes (Kikuchi et al. 2004; Galkina and Ley 2006). In a murine model of streptozotocin-induced diabetes, CX3CR1 deficiency decreased extracellular matrix deposition (Song et al. 2013). Clinical data may however suggest that rather CCL2 (MCP-1), if any single cytokine, is mechanistically required in diabetic kidney disease, at least in humans (Menne et al. 2017).

### Ischemia reperfusion injury

There is conflicting data on a mechanistic role of CX3CR1 in experimental ischemia-reperfusion injury (IRI), reporting both deleterious (Li et al. 2008) and protective functions (Oh et al. 2008).

An interesting aspect to non-renal modulators of the severity of renal IRI was published in 2017: depletion of gut microbiota by broad-spectrum antibiotic therapy decreased abundance of renal CX3CR1^+^ cells already in the absence of injury (Emal et al. 2017). Similar reductions were observed for other myeloid cell markers such as CCR2 and F4/80, while the mean CX3CR1 expression level on CX3CR1^+^ cells was unaffected. Cell depletion was reversible after fecal transplantation. After ischemia reperfusion injury, renal damage was significantly reduced in these mice. This proposes to investigate a possible impact of a plastic CX3CR1^+^ cell phenotype remains in this condition.

### Septic kidney injury

During acute kidney injury in polymicrobial sepsis, a combination of murine and human studies demonstrated worse outcome in mice lacking CX3CR1 (Chousterman et al. 2016). This was attributed to protective adhesion of classical monocytes to the vascular endothelium via this CX3CR1. In humans, the same study showed that individuals carrying one of the known common CX3CR1 genetic polymorphisms that enhance cell adhesion were found to have a lower incidence of septic acute kidney injury. This suggests a protective role for CX3CR1 in septic acute kidney injury.

### Infection

The CX3CR1-CX3CL1 axis plays a differential role in renal infection. The lack of CX3CR1 was irrelevant for host-response in pyelonephritis caused by *Escherichia coli*, at least 1 day after infection (Hochheiser et al. 2013). On the other hand, it determined survival in systemic candidiasis via resolution of kidney infection (Lionakis et al. 2013). This was attributed to the role of CX3CR1 in renal myeloid phagocyte homing. Consistently, assessment of human CX3CR1 polymorphisms suggested that it is required in renal (Lionakis et al. 2013), but not mucosal, anti-candidal defense in humans (Break et al. 2015). These findings suggested a kidney-specific role for CX3CR1 in the antifungal defense.

### Kidney injury induced by radio-contrast agents

Another recent study addresses myeloid cells in kidney injury induced by radio-contrast agents (Lau et al. 2018). CX3CR1^+^ phagocytes were central for contrast agent uptake, which occurred within minutes. For ensuing damage, the NLRP3 inflammasome was required, since IL-1β blockade was protective. The impact of the myeloid cells, but not necessarily monocytes, was demonstrated by decreased damage after anti-Ly6G treatment and, more specifically for phagocyte impact, by a beneficial effect of liposomal clodronate, which depletes these cells. It was not tested whether CX3CR1 was mechanistically involved, but such a role appears likely given its major role in renal homing.

### Fibrotic renal conditions

There are conflicting data on a mechanistic impact of CX3CR1 in renal fibrosis. While some studies suggest profibrotic action after ischemia reperfusion injury (Furuichi et al. 2006) and more recently, after oral fructose treatment (Yu et al. 2018), both pro- and antifibrotic effects of genetic CX3CR1 deletion were reported in unilateral ureteral obstruction in mice (Engel et al. 2015; Peng et al. 2015). Also in kidney fibrosis induced by hypertension, while an earlier report describes aggravation by CX3CR1 in DOCA salt–treated mice (Shimizu et al. 2011), a more recent study found less renal hypertensive damage in unilaterally nephrectomized angiotensin II-infused mice on a high salt diet in the presence of CX3CR1 than in its absence (Ahadzadeh et al. 2018). There was no difference in cardiac fibrosis. Some of the differences may be attributable to the use of older mice and a later histological endpoint in the first study.

Newer data from a different organ system, namely, the lung, may contribute to the understanding of the CX3CL1-CX3CR1 system in fibrosis. After demonstrating local and systemic CX3CL1 upregulation in human pulmonary fibrosis, the authors continued with functional studies of human monocytes from patients and controls. Here, they found differential effects of endothelial CX3CL1 with a pro-migratory effect on patient, but not control cells (Greiffo et al. 2020). The underlying mechanisms of differential monocyte response remain to be elucidated. They may be relevant also for patients with kidney disease.

### Systemic CX3CR1^+^ effects with potential impact on kidney disease

CX3CR1^+^ cells are also abundant in tissues lining the intestine. In their absence, the integrity of the intestinal barrier was compromised, resulting in an altered microbiome, endotoxemia in the portal blood, and aggravation of inflammatory conditions of the gut and liver (Schneider et al. 2015; Kim et al. 2018). It is conceivable that such proinflammatory effects may also reach the kidney.

A recent publication addresses the role of CX3CR1^+^ cells in induction of tertiary lymphoid structures and IgA response in salmonella colitis and determined a mechanistic requirement of the CX3CR1^HIGH^ expressing subtype (Koscsó et al. 2020). However, while CX3CR1-promotor–driven Cre-recombinases were used to assess the role of antigen presentation by this cell type, the study did not address the functional role of the CX3CR1 molecule itself in this process. Still, the most common glomerulonephritis worldwide, IgA nephropathy, often coincides by inflammatory bowel disease and/or is triggered by inflammatory episodes there. Therefore, CX3CR1^+^ cells in the intestine and possibly their renal homing, or the homing of T cells activated by them, may be tested as a mechanistic link between both diseases.

Another recently described systemic effect of CX3CR1 is dead cell disposal. An experiment addressing muscle cell death induced by influenza virus infection demonstrates that CX3CR1+ cells are required for phagocytic disposal (Runyan et al. 2020). Similarly, phagocytic disposal was impaired in 20–24 months old aged mice. This finding may be important in other tissues with limited regeneration potential including the kidney.

## Concluding remarks

The CX3CL1-CX3CR1 ligand-receptor pair plays a complex role in kidney disease and continues to raise interest in the treatment of vascular complications and in patients with kidney disease (Low et al. 2020). Human expression data and experimental models consistently support a pathophysiologic role of CX3CL1/CX3CR1 in inflammatory processes in the renal cortex such as glomerulonephritis and transplant rejection. Effects in predominantly fibrotic disease are less well understood on the mechanistic level. Systemic effects of the CX3CL-CX3CR1 system have been demonstrated in the gut. These may also be relevant for remote effects of renal inflammation and failure.

In the future, emerging technologies such as single-cell mRNA sequencing may allow better understanding of expression and gene regulation of the CX3CL-CX3CR1. As mRNA does not always correlate with functional receptor surface expression in terminally differentiated migratory cells, novel technological platforms for spatially resolved, multiplexed immunofluorescence may further our understanding by providing high-dimensional proteomic information on the single-cell level. Such information may close the gaps in our understanding of the complex biology of the CX3CL-CX3CR1 axis and its unique role in renal disease, thereby opening new avenues for more specific therapies of kidney disease.
